# Rare Tumors Presenting as a Mastoid Mass

**DOI:** 10.1155/2020/8985730

**Published:** 2020-06-07

**Authors:** Jiyeon Lee, Kyujin Han, Chang-Hee Kim

**Affiliations:** Department of Otorhinolaryngology-Head and Neck Surgery, Konkuk University Medical Center, Research Institute of Medical Science, Konkuk University School of Medicine, Seoul, Republic of Korea

## Abstract

In terms of diagnosis, a painless isolated mass lesion around the mastoid area is rarely encountered in general practice. In the present study, we report two cases of painless benign mastoid tumors located in the postauricular region. The first patient visited our department with a painless progressing mass lesion behind the right ear, which was later revealed as an intramuscular lipoma in the mastoid origin site of the sternocleidomastoid muscle. The second patient similarly presented to our department with a chief complaint of a painless, palpable mass in the mastoid region. Biopsy results confirmed the diagnosis of an osteoma. In both cases, the tumor was surgically removed by a postauricular approach. Although osteoma and lipoma are benign tumors rarely involved in the mastoid area, presenting without symptoms, it is recommended of complete excision, especially in cases with symptoms or cosmetic deformity.

## 1. Introduction

Subcutaneous lipomas are known to be the most common benign soft tissue tumors. They usually consist of mature fat cells which are encapsulated by thin layers of fibrous capsules. While lipomas are well encapsulated histologically in most cases, the intramuscular type, which accounts for 1.9% of all lipomas, arises between muscle fibers and penetrates through the intramuscular septa [1]. Intramuscular lipomas are known to be rarely seen in the head and neck region, but they are most commonly located in the extremities, chest wall, or abdomen [2–4].

Osteomas are histologically composed of mature compact bone with predominant laminar structure, and reports of mastoid osteomas are also known to be scarce. They are usually asymptomatic and develop slowly, presenting later on as a cosmetic deformity such as an external mass or auricular protrusion [5, 6].

Here, we report two rare cases of patients with postauricular masses who were diagnosed with an intramuscular lipoma and a mastoid osteoma, respectively.

## 2. Case Presentation

### 2.1. Case 1

**Figure 1:**
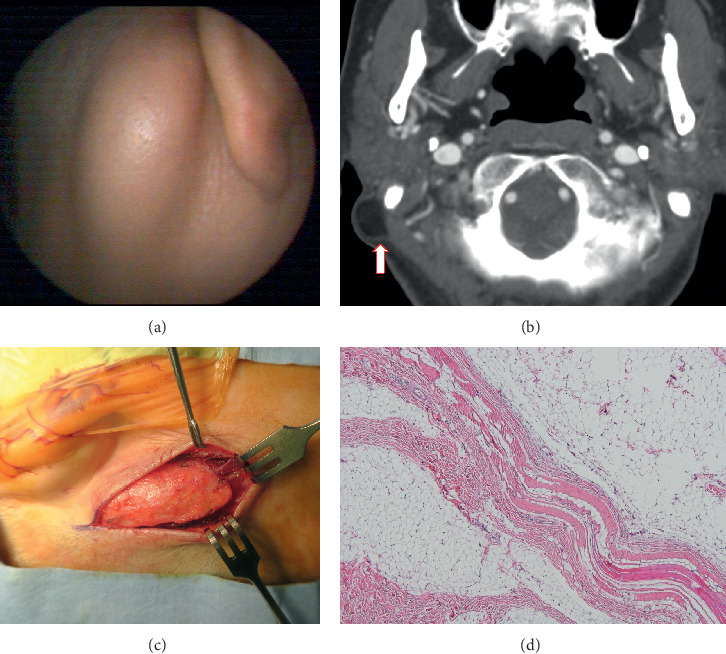
Right postauricular bulging mass which is asymptomatic, soft, and slightly movable (a). The temporal bone computed tomography shows a well-defined, low-attenuated, and nonenhancing mass (arrow) lateral to the mastoid process (b). Intraoperative view of a well-circumscribed intramuscular benign lipoma (c). A benign lipoma surrounded by muscle fibers. The tumor was composed of mature adipocytes without lipoblasts or cytologic atypia (d) (H&E × 100).

A 60-year-old female patient visited our department with a complaint of a painless and slowly growing mass on the right postauricular area. The physical examination of the patient revealed a slightly movable and oval-shaped mass with soft and smooth margin (Figure 1(a)). It was located at the mastoid tip area beneath the sternocleidomastoid (SCM) muscle insertion site. The computed tomography (CT) revealed a well-circumscribed 4.4 × 1.3 cm sized mass, with an adipose tissue signal density beneath the mastoid insertion site of the right SCM muscle (Figure 1(b)). The surgical removal was performed under local anesthesia, and the well-circumscribed lipomatous mass was noted between the SCM muscle fibers (Figure 1(c)). It is emphasized that the tumoral mass was poorly circumscribed partly in the medial surface, and it was dissected bluntly and excised totally without any complication. The histological examination demonstrated that the tumor consisted of mature fat cells, without lipoblasts or any sign of cytologic atypia. Some of lipocytes were infiltrated into the striated muscle tissue (Figure 1(d)). The postoperative course was uneventful, and no recurrence was seen in the 41 months of follow-up.

### 2.2. Case 2

**Figure 2:**
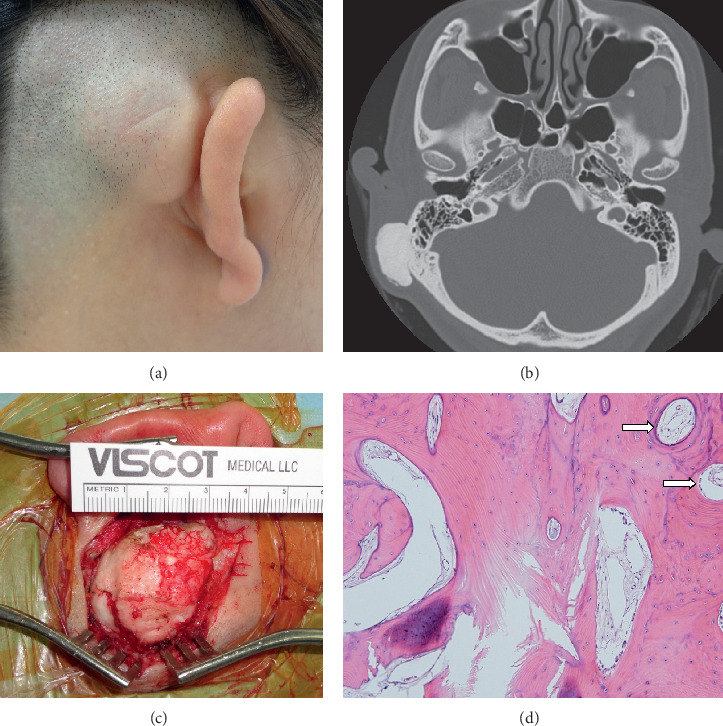
Right postauricular bulging mass which is asymptomatic, hard, and non-movable (a). Temporal bone computed tomography shows a huge, well-demarcated, and bony mass on the right mastoid cortex (b). The intraoperative view of a large bony tumor after elevation of the periosteum (c). The microscopic finding showing areas composed primarily of compact, mature, lamellar bone with organized Harvesian canals (d) (arrows, H&E  × 100).

A 44-year-old female patient visited our clinic with a complaint of a slowly growing, hard mass on the right postauricular area, which had been noticed since 10-years timeframe prior to this visit. On a physical examination, a 3 × 3 cm, hard, smooth, painless, and unmovable mass in the mastoid area was observed (Figure 2(a)). The temporal bone CT showed that a 2.3 × 1.4 × 3.0 cm sized outgrowing osteoma with irregular surface was observed in the right mastoid cortex (Figure 2(b)). The resection of tumor was performed via a retroauricular approach under general anesthesia (Figure 2(c)). The peduncle of the osteoma was excised with an oscillating saw, and then the margin of the mass was drilled out completely with a diamond burr. The histologic findings showed a dense compact lamellar bone, consistent with a compact osteoma (Figure 2(d)). The postoperative course was uneventful, and no recurrence was seen in 36 months of follow-up.

## 3. Discussion

For the differential diagnosis of postauricular masses, hemangioma, lipoma, dermoid cyst, epidermoid cyst, and trichilemmal cyst are to be considered [7]. Most of the cases are known to be benign in nature, asymptomatic, and may cause cosmetic deformity if not properly treated.

Intramuscular lipomas arising from the SCM are known to be very rear [2–4, 8]. Common histological characteristics include mature fat cells with sparse blood vessels and incomplete fibrous capsule covering the tumor with scattered muscle bundles within the adipose tissue. They are clinically presenting as slowly growing, painless, and round-shaped tumorous lesions. Although the exact pathogenesis remains unclear, factors such as obesity, metaplasia, trauma, chronic irritation, and congenital development have been suggested. Due to its infiltrative features, it is essential for the physician to distinguish intramuscular lipomas from well-differentiated liposarcomas. A preoperative CT demonstrates the characteristic low attenuation, even though infiltrative margins are not easily identified by a CT scan [9]. There have been reports of infiltrative intramuscular lipomas showing high recurrence rates [1], and a wide excision is recommended to prevent recurrence.

Osteoma in the temporal bone, also a scarcely reported entity, comprises 0.1 to 1% of all benign tumors of the cranial bone [10]. Osteomas that originate from the mastoid cortex are usually known to present without a symptom, unless the patients complains of cosmetic deformity at the point when it grows enough to be noticed. Otherwise, the enlarged osteomas may protrude through the external ear, interfering when wearing glasses, and cause local tenderness [11]. A temporal bone CT is the most appropriate modality for diagnosis which reveals characteristics of a dense, well-demarcated, outgrowing bony mass from the mastoid cortex. Although superficial mastoid osteomas rarely extend medially into the petrous bone, there are reported cases of intrapetrosal extension adjacent to the facial nerve, lateral semicircular canal, or ossicles [12–14]. Differential diagnosis is required to distinguish from other mastoid bone tumors including osteosarcoma, giant cell tumor, bone metastasis, multiple myeloma, fibrous dysplasia, and Paget's disease [11]. Histologically, the osteoma is characterized as a mesenchymal osteoblastic tumor possessing well-differentiated mature osseous tissue with a laminar structure and is typically divided into three subtypes: compact, cancellous, and mixed [5, 6, 10, 11]. Treatment includes surgical excision of the tumor with sufficient drilling of the tumor base until the normal temporal bone is identified to ensure complete removal of the tumor.

## 4. Conclusions

Intramuscular lipoma and osteoma are considered to be rare entities that should not be overlooked in differential diagnosis for a postauricular mass. Identifying the correct type of lesion is critical, and a thorough preoperative clinical and radiological evaluation is essential to avoid recurrence after surgical excision.
